# Pregnancy intervals after stillbirth, neonatal death and spontaneous abortion and the risk of an adverse outcome in the next pregnancy in rural Bangladesh

**DOI:** 10.1186/s12884-019-2203-0

**Published:** 2019-02-09

**Authors:** Bareng A. S. Nonyane, Maureen Norton, Nazma Begum, Rasheduzzaman M. Shah, Dipak K. Mitra, Gary L. Darmstadt, Abdullah H. Baqui, for the Projahnmo Study Group in Bangladesh

**Affiliations:** 1 Department of International Health and International Center for Maternal and Newborn Health, Johns Hopkins Bloomberg School of Public Health, 615 N. Wolfe Street, Baltimore, MD 21205 USA; 2 Bureau for Global Health, Office of Population and Reproductive Health, USAID, Washington D.C, USA; 3 School of Public Health, Independent University Bangladesh (IUB), Dhaka, Bangladesh; 4 March of Dimes Prematurity Research Center, Division of Neonatology, Department of Pediatrics, Stanford University School of Medicine, Stanford, CA USA

**Keywords:** Pregnancy spacing, inter-outcome intervals, stillbirth, neonatal death, spontaneous abortion, adverse pregnancy outcomes

## Abstract

**Background:**

Studies have revealed associations between preceding short and long birth-to-birth or birth-to-pregnancy intervals and poor pregnancy outcomes. Most of these studies, however, have examined the effect of intervals that began with live births. Using data from Bangladesh, we examined the effect of inter-outcome intervals (IOI) starting with a non-live birth or neonatal death, on outcomes in the next pregnancy. Pregnancy spacing behaviors in rural northeast Bangladesh have changed little since 2004.

**Methods:**

We analyzed pregnancy histories for married women aged 15-49 years who had outcomes between 2000 and 2006 in Sylhet, Bangladesh. We examined the effects of the preceding outcome and the IOI length on the risk of stillbirth, neonatal death and spontaneous abortion using multinomial logistic regression models.

**Results:**

Data included 64,897 pregnancy outcomes from 33,495 mothers. Inter-outcome intervals of 27-50 months and live births were baseline comparators. Stillbirths followed by IOI’s <=6 months, 7-14 months or overall <=14 months had increased risks for spontaneous abortion with adjusted relative risk ratios (aRRR) and 95% confidence intervals = 29.6 (8.09, 108.26), 1.84 (0.84, 4.02) and 2.53 (1.19, 5.36), respectively. Stillbirths followed by IOIs 7-14 months had aRRR 2.00 (1.39, 2.88) for stillbirths.

Neonatal deaths followed by IOIs <=6 months had aRRR 28.2 (8.59, 92.63) for spontaneous abortion. Neonatal deaths followed by IOIs 7-14 and 15-26 months had aRRRs 3.08 (1.82, 5.22) and 2.32 (1.38, 3.91), respectively, for stillbirths; and aRRRs 2.81 (2.06, 3.84) and 1.70 (1.24, 3.84), respectively, for neonatal deaths.

Spontaneous abortions followed by IOIs <=6 months and 7-14 months had, respectively, aRRRs 23.21 (10.34, 52.13) and 1.80 (0.98, 3.33) for spontaneous abortion.

**Conclusion:**

In rural northeast Bangladesh, short inter-outcome intervals after stillbirth, neonatal death and spontaneous abortion were associated with a high risk of a similar outcome in the next pregnancy. These findings are aligned with other studies from Bangladesh. Two studies from similar settings have found benefits of waiting six months before conceiving again, suggesting that incorporating this advice into programs should be considered. Further research is warranted to confirm these findings.

**Electronic supplementary material:**

The online version of this article (10.1186/s12884-019-2203-0) contains supplementary material, which is available to authorized users.

## Background

Several studies have shown that short intervals between pregnancies are associated with subsequent poor pregnancy outcomes, including spontaneous abortion, stillbirth, preterm birth, low birth weight (LBW), congenital malformations, small for gestational age (SGA), and early neonatal death [1–16]. Similarly, long inter-pregnancy intervals (IPIs) greater than 59 months have been associated with adverse perinatal outcomes, namely, preterm birth, LBW, and SGA. [14, 15, 17]. Most of these studies focus on intervals beginning with live births.

Globally, 122 million live births occur annually and of these, 2.7 million result in neonatal deaths. In addition, women experience an estimated 28 million spontaneous abortions and 2.6 million stillbirths [18, 19]. In 2005, experts at a World Health Organization (WHO) technical consultation recommended that women should wait at least 24 months after a live birth before conceiving again [20]. Approximately, a 24 month live birth-to-pregnancy interval is equivalent to a 33 month birth-to-birth interval. It is, however, recognized that this recommendation is difficult to follow when parents have just experienced a loss. More work is needed on ways to provide emotional support and guidance to families.

To date, researchers have focused little attention on the effect of intervals after non-live birth outcomes. In Matlab, Bangladesh, research on the effect of the interval between a live birth, or a non-live birth, and a subsequent outcome showed that the risk of under-five mortality varied by the type of outcome that started the interval [21]. Another study in Bangladesh that examined three non-live birth outcomes (miscarriage, induced abortion and stillbirth) found that inter-pregnancy intervals that began with a non-live birth outcome were generally more likely to end with the same type of non-live birth outcome than those that began with a live birth [22]. The authors noted that their study area (Matlab) “has access to unusually good maternal and child health care and family planning services.” They urged that similar studies be conducted in communities in low income countries.

Across Bangladesh, intervals after live births have generally improved over time but Sylhet has been lagging behind other districts [23, 24]. It had the lowest median birth interval of 38 months in 2011, and 36.7 months in 2014. For the rest of the country’s districts, the median birth interval rose from 39 in 2004 to 47 months in 2011, and 57 months in 2014. Furthermore in 2011, 46.5% of non-first births in Sylhet occurred after an interval of 36 months (18.6 % within 24 months), increasing to 48.5% in 2014 (17.3% within 24 months).

Underscoring the need to counsel women who have experienced a neonatal death, the 2014 Bangladesh Demographic and Health Survey observed that “the length of the birth interval is closely associated with the survival status of the previous sibling.” In Bangladesh, among various sub-groups identified in the 2014 DHS, the highest percentages of very short interval births (defined as less than 18 months since the preceding birth) occurred in the group in which the preceding sibling had died (23%), compared to short interval births which occurred when the sibling was alive (3%), or in the group of women in the lowest income quintile (5%) or rural women (5%). Having a preceding death was associated with short IPIs reflecting the fact that parents who have lost a child wish to have another one shortly thereafter to replace the one who has died [23, 24].

Risk factors for adverse perinatal outcomes and the clustering of neonatal deaths in the Sylhet cohort studied in this analysis have been described [25, 26] but few investigations have been conducted on the combined effects, on an index pregnancy outcome, of the length of the inter-pregnancy interval [22] and a prior adverse birth outcome (stillbirth, neonatal death, and spontaneous abortion). Our analysis uses inter-outcome intervals (IOIs) from pregnancy history recall to try to address this gap in the literature related to this rural Bangladesh setting.

## Study design and methods

The data were drawn from a study known as ‘Projahnmo I’ which was a community-based, cluster-randomized trial conducted in the Sylhet district of Bangladesh. Its aim was to evaluate the effect of an integrated maternal and newborn care program on neonatal mortality, and the results have been reported elsewhere [27]. Briefly, the trial had 3 arms, namely, a comparison area with usual care; a home-based care arm where trained community health workers (CHWs) made home visits to provide counselling during pregnancy and the immediate post-partum period; and a community care arm where health promotion was conducted through group sessions. Each arm was made up of 8 clusters of population sizes around 20,000. We used data from the endline survey, which had been carried out by specially trained data collectors in 2006, to determine whether interventions resulted in changes in neonatal mortality rates and maternal and newborn care practices. The survey respondents were married women aged 15-49 years who had had at least one pregnancy outcome between 2000 and 2006.

The Projahnmo I trial was registered with ClinicalTrials.gov NCT00198705.

### Defining the intervals

Data on intervals between non-live birth outcomes in low income countries are not widely available. For example, the Demographic and Health Surveys (DHS) do not routinely gather information on birth intervals, or pregnancy outcomes, after miscarriage, stillbirth, or newborn death as a separate category. DHS data are available by survival status of the preceding sibling, a category that includes siblings of all ages who died.

Using Projahnmo data on pregnancy histories, we studied the following outcomes: stillbirth, neonatal death, and spontaneous abortion. In these data, we only had self-reported dates of pregnancy outcomes and thus we used the term inter-outcome interval (IOI), following DaVanzo et al. [21], who defined an IOI as the time period between the date of the outcome of the index pregnancy and the date of the outcome of the preceding pregnancy, even if one or both of these pregnancies had a non-live birth outcome. In comparison, a 14 month inter-outcome interval is the approximate equivalent of a 5 month inter-pregnancy interval, and an 18 month inter-outcome interval is the approximate equivalent of a 9 month inter-pregnancy interval.

We considered the following categories of the IOI which have been used in studies of IPI: <6, 7-14, 15-26, 27-50, and 51-74 and 75+ months, with 27-50 months used as a baseline comparator [21, 28] [17, 21, 22, 29]. The 27-50 months interval is often associated with relatively lower adverse outcomes and in our study it has the largest samples size

### Definition of outcomes and denominators

This analysis included pregnancies with self-reported outcomes and IOIs. We excluded women with only a first pregnancy in order to focus on the impact of the interval following an adverse pregnancy outcome. Neonatal deaths were defined as live births that subsequently resulted in mortality within the first 28 days after birth. The denominator for neonatal mortality included all live births. Stillbirths are clinically defined as those babies born with no signs of life at or after 28 weeks' gestation (i.e., who did not breathe or move after birth) [30] and in this study we relied on the women’s recall to categorize outcomes as stillbirths. The denominator for these was all births in our study. For the abortions outcome, only those that were reported as spontaneous rather than induced were included. The denominator for these was all pregnancies irrespective of type of outcome.

### Statistical Analysis

Our analysis was stratified by the type of outcome that started the interval (stillbirth, neonatal death and spontaneous abortion). Thus, we fitted three separate models so that, conditional on the interval being preceded by an adverse outcome, we evaluated the effect of the length of IOI on the subsequent outcome. Analogous to the analysis by DaVanzo et al 2007, we fitted multinomial logistic regression models where the 4-category multinomial outcome comprised live birth (the reference outcome), stillbirth, neonatal death and spontaneous abortion. The measures of association were thus risk ratios of the different IOIs (with 27-50 months taken as a reference) for adverse outcomes, relative to the risk ratios of these intervals for a live birth outcome. Hence they are relative risk ratios. Covariates adjusted for included multiple/single pregnancy; mother’s education, parity at current birth, and maternal age at current birth; household socio-economic status (SES) index; Projahnmo study arm; whether the mother attended at least one antenatal care visit, and place of birth (home versus facility). The SES index was derived from the household variables using principal components analysis [31]. The household variables were drinking water source; toilet type; availability of electricity; ownership of a wardrobe, table, radio, television, refrigerator, mobile phone, bicycle, car or tempo; and types of roof, wall and floor. Stata 2013 (StatCorp, College Station, Texas, USA) was used to conduct all analyses.

## Results

Our data included 64,897 pregnancy outcomes from 33,495 mothers. Table 1 provides characteristics of the mother, household, and child at the time of each pregnancy outcome. Table 2 summarizes index pregnancy outcomes by the preceding outcomes stratified by the length of the interval between these outcomes. This table illustrates a possible bias in the recall of dates of outcomes as there are reportedly stillbirths and neonatal deaths occurring within 6 months, which is not possible. It shows that spontaneous abortions made up 44 - 71% of the index outcomes within the <=6 months IOI, across preceding outcomes of various types. In general, across all intervals, adverse outcomes were more likely when preceded by one of a similar type.

**Table 1:** Baseline characteristics

	N	%
Total	64,897	100
Study Arm		
1	20,767	32.00
2	22,740	35.04
3	21,390	32.96
Mothers' education		
no school	33,105	51.01
1-5 years	19,038	29.34
6+ years	12,754	19.65
Birth type		
Single	59,610	91.85
Multiple	1,229	1.89
Unknown*	4,058	6.25
Mother's age at outcome		
<20	1,649	2.54
20-<=25yrs	14,169	21.83
>25-<=30yrs	23,292	35.89
>30	25,787	39.74
Child sex		
1	30,763	47.40
2	29,717	45.79
Unknown*	4,417	6.81
Household wealth quintile		
Most poor	14,014	21.59
2	13,159	20.28
3	12,752	19.65
4	12,939	19.94
Least poor	12,033	18.54

*May not be applicable depending on the outcome type

**Table 2:** The numbers (and percentages in the lower row) of index outcomes by the preceding outcome, stratified by the IOI

IOI<=6 months ^a^					
Preceding outcome	Index outcome				
	Live	Spontaneous abortion	Stillbirth	Neonatal death	Total
Spontaneous abortion	63	173	4	4	*244*
	25.82	70.9	1.64	1.64	*100*
Stillbirth	20	23	0	1	*44*
	45.45	52.27	0	2.27	*100*
Neonatal death	27	32	6	7	*72*
	37.5	44.44	8.33	9.72	*100*
Alive	76	152	12	12	*252*
	30.16	60.32	4.76	4.76	*100*
Total	*186*	*380*	*22*	*24*	*612*
	*30.39*	*62.09*	*3.59*	*3.92*	*100*
^a^Outcomes other than spontaneous abortion within 6 months reflect errors in recall of outcome dates					
IOI 7-14 months					
Preceding outcome	Index outcome				
	live	Spontaneous abortion	Stillbirth	Neonatal death	Total
Spontaneous abortion	1333	342	68	98	*1841*
	72.41	18.58	3.69	5.32	*100*
Stillbirth	585	66	123	54	*828*
	70.65	7.97	14.86	6.52	*100*
Neonatal death	896	68	68	207	*1239*
	72.32	5.49	5.49	16.71	*100*
Alive	3,573	721	232	370	*4896*
	72.98	14.73	4.74	7.56	*100*
Total	*6387*	*1197*	*491*	*729*	*8804*
	*72.55*	*13.6*	*5.58*	*8.28*	*100*
IOI 15-26 months					
Preceding outcome	Index outcome				
	live	Spontaneous abortion	Stillbirth	Neonatal death	Total
Spontaneous abortion	1254	183	49	68	*1554*
	80.69	11.78	3.15	4.38	*100*
Stillbirth	734	53	110	63	*960*
	76	5.52	11.46	7	*100*
Neonatal death	1205	61	71	168	*1505*
	80.07	4.05	4.72	11.16	*100*
Alive	16,867	1123	474	745	*19,209*
	87.81	5.85	2.47	3.88	*100*
Total	*20,132*	*1421*	*711*	*1090*	*23,354*
	*86.2*	*6.08*	*3.04*	*4.67*	*100*
IOI 27-50 months					
Preceding outcome	Index outcome				
	live	Spontaneous abortion	Stillbirth	Neonatal death	Total
Spontaneous abortion	568	68	25	24	*685*
	82.92	9.93	3.65	3.5	*100*
Stillbirth	412	28	47	26	*513*
	80.31	5.46	9.16	5.07	*100*
Neonatal death	722	34	19	59	*834*
	86.57	4.08	2.28	7.07	*100*
Alive	20,386	869	544	740	*22,539*
	90.45	3.86	2.41	3.28	*100*
Total	*22,186*	*1000*	*656*	*884*	*24,726*
	*89.73*	*4.04*	*2.65*	*3.58*	*100*
IOI 51-74 months					
Preceding outcome	Index outcome				
	live	Spontaneous abortion	Stillbirth	Neonatal death	Total
Spontaneous abortion	109	16	9	6	*140*
	77.86	11.43	6.43	4.29	*100*
Stillbirth	80	3	10	5	*98*
	81.63	3.06	10.2	5.1	*100*
Neonatal death	140	7	4	6	*157*
	89.17	4.46	2.55	3.82	*100*
Alive	4401	249	158	162	*4970*
	88.55	5.01	3.18	3.26	*100*
Total	*4756*	*275*	*185*	*190*	*5406*
	*87.98*	*5.09*	*3.42*	*3.51*	*100*
IOI 75-263 months					
Preceding outcome	Index outcome				
	live	Spontaneous abortion	Stillbirth	Neonatal death	Total
Spontaneous abortion	34	7	1	2	*44*
	77.27	15.91	2.27	4.55	*100*
Stillbirth	35	2	4	3	*44*
	79.55	4.55	9.09	6.82	*100*
Neonatal death	57	8	7	5	*77*
	74.03	10.39	9.09	6.49	*100*
Alive	1874	129	73	76	*2152*
	87.08	5.99	3.39	3.53	*100*
Total	*2019*	*146*	*85*	*90*	*2340*
	*86.28*	*6.24*	*3.63*	*3.85*	*100*

Table 3 shows the unadjusted and adjusted relative risk ratios (aRRR) and confidence intervals (CI) of each of the adverse outcomes due to the length of the IOI, stratified by preceding outcome.

**Table 3:** Unadjusted and adjusted Relative Risk Ratios^1^

Preceding outcome=spontaneous abortion								
Index outcome	Unadjusted				Adjusted			
	Relative risk ratio		Lower CI	Upper CI	Relative risk ratio		Lower CI	Upper CI
Live birth	Reference outcome							
Spontaneous abortion								
<=6 months	22.94		15.64	33.63	23.21		10.34	52.13
7-14 months	2.14		1.62	2.83	1.80		0.98	3.33
15-26 months	1.22		0.91	1.64	1.05		0.55	2.01
27-50 months	Reference							
51-74 months	1.23		0.69	2.19	0.33		0.11	0.98
75-263 months	1.72		0.73	4.03	0.28		0.05	1.59
Stillbirth								
<=6 months	1.44		0.49	4.28	1.36		0.45	4.07
7-14 months	1.16		0.73	1.85	1.05		0.65	1.69
15-26 months	0.89		0.54	1.45	0.80		0.49	1.32
27-50 months	Reference							
51-74 months	1.88		0.85	4.13	1.66		0.74	3.74
75-263 months	0.67		0.09	5.08	0.73		0.09	5.68
Neonatal death								
<=6 months	1.50		0.51	4.47	1.39		0.46	4.16
7-14 months	1.74		1.10	2.75	1.55		0.97	2.46
15-26 months	1.28		0.80	2.07	1.17		0.72	1.90
27-50 months	Reference							
51-74 months	1.30		0.52	3.26	1.09		0.42	2.82
75-263 months	1.39		0.32	6.14	1.20		0.26	5.61
Preceding outcome=Stillbirth								
Index outcome	Unadjusted				Adjusted			
	Relative risk ratio		Lower CI	Upper CI	Relative risk ratio		Lower CI	Upper CI
Live birth	Reference outcome							
Spontaneous abortion								
<=6 months	16.92		8.31	34.45	29.60		8.09	108.26
7-14 months	1.66		1.05	2.63	1.84		0.84	4.02
15-26 months	1.06		0.66	1.71	0.76		0.35	1.65
27-50 months	Reference							
51-74 months	0.55		0.16	1.86	0.36		0.07	2.00
75-263 months	0.84		0.19	3.68	0.82		0.09	7.44
Stillbirth								
<=6 months	^2^─	─	─	─	─	─	─	─
7-14 months	1.84		1.29	2.64	2.00		1.39	2.88
15-26 months	1.31		0.91	1.89	1.33		0.92	1.91
27-50 months	Reference							
51-74 months	1.10		0.53	2.26	1.12		0.54	2.34
75-263 months	1.00		0.34	2.94	1.02		0.34	3.02
Neonatal death								
<=6 months	0.79		0.10	6.14	0.82		0.10	6.42
7-14 months	1.46		0.90	2.37	1.44		0.88	2.37
15-26 months	1.36		0.85	2.18	1.29		0.79	2.09
27-50 months	Reference							
51-74 months	0.99		0.37	2.66	0.99		0.36	2.73
75-263 months	1.36		0.39	4.71	1.34		0.37	4.76
Preceding outcome=Neonatal Death								
Index outcome	Unadjusted				Adjusted			
	Relative risk ratio		Lower CI	Upper CI	Relative risk ratio		Lower CI	Upper CI
Live birth	Reference outcome							
Spontaneous abortion								
<=6 months	25.17		13.58	46.64	28.20		8.59	92.63
7-14 months	1.61		1.06	2.46	1.97		1.00	3.89
15-26 months	1.07		0.70	1.65	1.59		0.81	3.12
27-50 months	Reference							
51-74 months	1.06		0.46	2.44	1.05		0.27	4.06
75-263 months	2.98		1.32	6.74	1.97		0.49	7.91
Stillbirth								
<=6 months	8.44		3.12	22.84	10.05		3.64	27.74
7-14 months	2.88		1.72	4.84	3.08		1.82	5.22
15-26 months	2.24		1.34	3.75	2.32		1.38	3.91
27-50 months	Reference							
51-74 months	1.09		0.36	3.24	1.02		0.33	3.11
75-263 months	4.67		1.88	11.57	4.01		1.55	10.33
Neonatal death								
<=6 months	3.17		1.33	7.59	3.22		1.33	7.80
7-14 months	2.83		2.08	3.84	2.81		2.06	3.84
15-26 months	1.71		1.25	2.33	1.70		1.24	2.33
51-74 months	0.52		0.22	1.24	0.54		0.23	1.28
75-263 months	1.07		0.41	2.78	1.14		0.43	3.01

^**1**^The reference IOI=27-50 months, reference outcome=live birth. Models adjusted for multiple/single pregnancy; mother’s education, parity at current birth, and maternal age at current birth; household socio-economic status (SES) index; Projahnmo study arm; whether the mother attended at least one antenatal care visit, and place of delivery (home versus facility )

^2^Too little data to get estimates; bias in recall of outcome dates

### Intervals starting with spontaneous abortion

Short (<=6 months) IOIs that began with a spontaneous abortion, were associated with relatively higher risks of another spontaneous abortion versus a live birth, compared to intervals of 27-50 months [aRRR 23.21 (CI: 10.34, 52.13)]. The interval 7-14 months was also associated with an increased risk of another spontaneous abortion [aRRR 1.8 (CI: 0.98, 3.33)]. When the intervals <=6 months and 7-14 months were merged to increase the sample size and to improve reliability of the estimates, the relative risk for spontaneous abortion versus a live birth remained strong in this IOI category versus 27-50 months [aRRR 2.70 (CI: 1.49,4.9)] (Additional file 1: Table S1)

Other intervals that began with a spontaneous abortion were not associated with higher risks of an adverse outcome, as compared to the 27-50 months interval.

### Intervals starting with stillbirth

Short (<=6 months) intervals that began with stillbirths, were associated with higher relative risks for a spontaneous abortion than a live birth, compared to births following IOIs of 27-50 months [aRRR 29.6 (CI: 8.09, 108.26)]. The estimated aRRR for the 7-14 months category was 1.84(CI: 0.84, 4.02). When using the combined <=6 months and 7-14months intervals, the aRRR was 2.53 (CI: 1.19, 5.36) for spontaneous abortion and 1.91(CI: 1.33, 2.75) for stillbirth.

### Intervals starting with neonatal death

Having had a neonatal death was associated with a higher risk of all three adverse outcomes versus live outcomes, for the IOIs shorter than 24 months relative to the 27-50 months reference IOI. The aRRR for spontaneous abortion was 28.2 (CI: 8.59, 92.63) for <=6 months compared to 27-50 months IOI. When the first two intervals (<=6 and 7-14 months) were combined, the associations remained strong with an aRRR of 2.82 (CI: 1.46, 5.45).

IOIs beginning with a neonatal death were associated with increased risks of stillbirth [<14 months aRRR 3.27 (CI: 1.94, 5.51), 15-26 months aRRR 2.32 (CI: 1.38, 3.91) and >75 months 4.01(CI: 1.55, 10.33)], and neonatal death [<14 months aRRR 2.82 (CI: 2.07,3.85), 15-26 months aRRR 1.70 (CI: 1.24,3.84)].

## Discussion

This study demonstrates that the highest risk for a spontaneous abortion was following another spontaneous abortion, or after a short IOI <=14 months irrespective of the preceding outcome. After a stillbirth, the IOIs <=14 months were associated with a higher risk of another stillbirth or a spontaneous abortion. Having a prior neonatal death was associated with an increased risk of another neonatal death or a non-live outcome for intervals up to 26 months relative to 27-50 months. The risk of a stillbirth after a neonatal death was also significantly higher for IOI’s >72 months, suggesting a U-shaped relationship where the risk decreased up to 27 months, but then increased again after 72 months. Overall, following an adverse outcome, a short IOI was associated with a risk of another adverse outcome in the subsequent pregnancy. Further research that appropriately measures gestational age and IPI is needed to help guide family planning interventions.

A recent study from rural Bangladesh has demonstrated that incorporating post-partum family planning into the maternal and newborn care packages can significantly improve birth spacing and reduce the risk of preterm birth [adjusted RR = 0.79; 95% CI = 0.63-0.99)] [32]. Given the relative lack of data from low income countries, it is understandable that there are few protocols for family planning counseling for women who have experienced non-live birth outcomes. The expert recommendation to WHO has been to wait six months after a spontaneous abortion before conceiving again [20]. The experts noted, however, that this recommendation was based on only one large study (n=258,108 women) in Latin America from two countries (Argentina and Uruguay) that used hospital records. This study was also unable to distinguish between spontaneous and induced abortions. Since then, a 2016 study in India by Chadna et al. has supported this recommendation [33]. This India study found that “women who conceive between 6-12 months of an initial miscarriage have better outcomes and lower complication rates in their subsequent pregnancy.”

Most of the available literature on the recommendation for how long to wait after an adverse event before conceiving again come from high income countries and focus on waiting time after a miscarriage. Some results from high income countries suggest that conception within a short interval (6 months) after an abortion does not result in adverse pregnancy outcomes [34–38]. A recent review of 16 studies also led to the same conclusion [39].

In contrast, results from low- and middle-income countries [Latin America, Bangladesh (Matlab), India[33]] and our current analysis of Bangladesh (Sylhet) indicates that short intervals after a stillbirth, neonatal death or spontaneous abortion, are associated with increased risk of adverse pregnancy outcomes. A 2010 study in Matlab, Bangladesh, also showed that the maternal mortality risk was “higher for pregnancies that ended in induced abortion, miscarriage, or stillbirth, compared to those that resulted in a live birth” (odds ratios 4.2, 2.0, and 17.4, respectively) [40]. The study concluded that improved management of these outcomes was needed in order to reduce the maternal mortality associated with them. A Bangladesh study [21], based on 125,720 births, found that “very short intervals (< 15 months) following stillbirths and miscarriages were associated with significantly increased risks of early neonatal mortality (RR =1.87, p<0.001, and RR=1.48, p<0.01, respectively)” compared with inter-outcome intervals of 36-59 months that followed pregnancies in which the infant was born alive. The study found that other intervals after pregnancies that began with non-live birth outcomes also were associated with significantly higher mortality risk and the and the effects “did not vary much by interval length.” [21]. The authors of this study recommended that women experiencing these types of outcomes be advised to wait at least six months before becoming pregnant again.

Another Bangladesh study, based on 10,453 pregnancies found that, compared with inter-pregnancy intervals (IPIs) of 6-12 months, pregnancies that were conceived <3 months after a miscarriage were more likely to result in a live birth [41]. However, the study also found that very short IPIs of <3 months following a miscarriage were associated with significantly higher risk of late neonatal mortality for the infant born at the end of the IPI (adjusted hazard ratio (HR) 1.74, 1.06 to 2.84). The Conde-Agudelo study [8] found that in Latin America, compared with post-abortion (spontaneous and induced) IPIs of 18-23 months, inter-pregnancy intervals shorter than 6 months were significantly associated with increased risk of low birth weight, very low birth weight, preterm delivery, very preterm delivery, and adverse maternal outcomes in the next pregnancy.

Somewhat in contrast to these findings, a study from Bangladesh, based on 66,759 pregnancy outcomes, found that “if the preceding pregnancy ended in a miscarriage or stillbirth, there is an elevated risk that the index pregnancy will end with the same outcome, regardless of the amount of time since the previous pregnancy ended” [22]. The differences in the results between studies conducted in higher and lower resource settings, as well as within low-resource settings, may be due to contextual differences such as the availability of good healthcare facilities which helps ensure that such high risk pregnancies are more closely monitored and cared for. For example, DaVanzo’s data were drawn from Matlab, Bangladesh, an area having higher quality care, compared to services in rural, northeast Bangladesh. Differences in nutritional status, risk of infections and genetic predispositions may also be important factors.

Most of the studies in the literature have investigated the effect of intervals defined as inter-pregnancy or inter-birth depending on the available data [2, 5, 9, 10, 14, 28, 42, 43]. Considering that we did not have data on gestational age, our intervals were defined according to DaVanzo et al. [21] who studied pregnancy spacing and infant and child mortality. Despite the difference in the target age groups, we have found similar results to DaVanzo et al. They undertook analysis with and without (i.e. using IOIs) having data on gestational age, and the results were similar. The shortest and longest intervals in our study were associated with higher risks of adverse perinatal outcomes.

Evidence of effect modification of the interval by maternal age has been found where the risk of preterm delivery for older mothers (aged >34 years) was lower at shorter IPIs (<11 months) but higher at longer IPI’s (11-23 months) in comparison to mothers aged 25-34 years [42]. In our analysis, we found no evidence of interaction between IPI and mother’s age. Our results have also provided further evidence of clustering of adverse outcomes of the same type within the same mother. An in-depth discussion of clustering of neonatal deaths in this study population was given previously [26], and a similar discussion in an Indian setting was given by Williams et al. [44].

### Mechanisms through which intervals affect outcomes

We identified two possible mechanisms that might explain how short intervals may affect pregnancy outcomes: folate depletion and vertical transmission of infection. We discuss these mechanisms because there is evidence in the literature regarding these mechanisms’ effects on perinatal outcomes (not just infant child outcomes) and there is also evidence on Bangladeshi women’s health conditions as they relate to these mechanisms.

Folate depletion has been hypothesized as a mechanism through which short IPIs are associated with adverse perinatal outcomes. If pregnancy occurs three to four months after a birth, before sufficient repletion of folate resources, the risk of another miscarriage may increase. A review of seventeen studies in low, middle and high income countries concluded that “strong evidence exists that folate depletion occurs in women during the first three to four months postpartum.”[45]. Studies in rural Bangladesh have found folate deficiency in early pregnancy and among married, nulliparous women [46, 47]. One study of over 11,000 women examined folate levels, miscarriage, and stillbirth, and found that, after adjustment for confounders, compared to women without supplemental folate intake, those in the highest category of intake had a reduced relative risk of spontaneous abortion of 0.80 (p=0.001 95% CI 0.71, 0.90) and reduced risk of stillbirth 0.55 (0.30, 1.00) (p=0.06) [48].

With respect to pregnancy intervals, one study found that mean erythrocyte and serum folate levels were significantly lower among women with short IPIs (6 months or less) compared to women with longer intervals of 18-24 months (erythrocyte p=0.002; serum folate p =0.00001) [49]. A second study did not find an association between IPI and folic acid deficiency[50]. However, this study examined only two categories of IPIs (≤30 and > 30 months), and did not examine shorter intervals such as less than 6, 6-11,12-17, 18-23, or 24-29 months.

A review of causal mechanisms concluded that there is evidence to support the hypothesis that folate depletion constitutes a hypothetical mechanism that explains the increased risk of adverse perinatal outcomes in women with short pregnancy intervals (43). However, this review focused on birth weight and small for gestational age outcomes, rather than neonatal death, stillbirth, and miscarriage.

With respect to vertical transmission of infections, women infected with bacterial, fungal, or viral organisms may harbor the organisms at a site from which the newly-conceived fetus can be infected. One study noted that, in theory, the risk of infection of the fetus could be higher for women with short intervals because they have less time to recover between pregnancies [45]. Studies have found high prevalence of reproductive tract and sexually transmitted infections in Bangladesh [51]. One analysis concluded that cytomegalovirus, the most frequent congenital infection globally, is endemic in Bangladesh [52, 53]. It found that among 420 pregnant Bangladeshi women, the prevalence of cytomegalovirus IgG antibody was 66.7% by the age of 15-20 years, and 71.4% in the age group of 26-30 years. Studies have found an association between primary cytomegalovirus infection, miscarriage and fetal, neonatal and infant deaths [54, 55]. One study of 3,461 women assessed the effect of the interval between births on the risk of congenital CMV infection [56]. It found that women who seroconverted between deliveries ≤ 24 months apart had a four-fold higher risk of delivering a congenitally infected baby than women who seroconverted between deliveries >24 months apart (OR, 4.3; 95% CI, 1.4-14.2). This study, however, did not adjust for confounding factors such as maternal age, race, and socioeconomic status.

### Study strengths and limitations

The first strength of our study is that our result*s* are based on a large sample size and the pregnancy history survey was conducted as part of a household survey rather than in a hospital setting. Thus, our study was not likely to suffer from selection bias resulting from sampling only those who seek care at formal institutions. Per criteria set forth for assessing the quality of observational studies on pregnancy spacing [28], we examined five categories of IOI; controlled for 10 potential confounders, and we had only 2.3% missing outcomes.

The main limitation of our study is that CHWs relied on pregnancy history recall rather than obtaining data from medical records. Thus there were no gestational ages which would have enabled us to investigate the inter-pregnancy interval effect. The errors in the recall of timing of events may explain why there are reported outcomes other than spontaneous abortion within the 6 months IOIs, which is not possible. There may also be some misclassification bias where early neonatal deaths were reported as stillbirths and the other way round, and similarly for spontaneous abortions and induced abortion. However, to reduce errors in recall of dates, the CHWs in our study carried a list of dates for historical events that happened in recent previous months or years and they asked mothers to use those dates as reference as well. Furthermore, a conversion calendar was used so that if women reported a date using a local calendar, a corresponding western calendar date could be recorded.

## Conclusion

Using pregnancy history recall and outcome dates, our findings address a significant gap in the literature on the combined effect of an adverse pregnancy outcome and the length of an interval between pregnancies or pregnancy outcomes, on the risk of another adverse outcome in a subsequent pregnancy. Our data suggest that, in rural northeast Bangladesh, the six-month pregnancy interval following any of three adverse events (stillbirth, neonatal death, and spontaneous abortion) was associated with the highest risk of a spontaneous abortion in the next pregnancy. Following a stillbirth, a less than 14 month inter-outcome interval (i.e., a < five month inter-pregnancy interval) was associated with another stillbirth or spontaneous abortion, and following a neonatal death, an inter-outcome interval less than 26 months (i.e.,< 17 month inter-pregnancy interval) was associated with all three adverse outcomes.

Studies from other low-income settings have found benefits of waiting six months before conceiving again. This suggests that incorporating this advice in programs should be considered, but further research is needed. A recent study from rural Bangladesh has demonstrated that incorporating post-partum family planning into the maternal and newborn care packages can significantly improve birth spacing and reduce the risk of preterm birth. Formative research in Sylhet, Bangladesh, has shown that even though there is an understanding of the benefits of healthy spacing, women are not able to make their own pregnancy spacing decisions [57]. Interventions that are suitable for the local culture and that take gender inequalities into account are needed. Such interventions should also take into account the parents’ strong desire to quickly replace the lost child.

There is also a need for additional research to identify genetic and other bio-markers that are associated with clustering of adverse outcomes within mothers as has been established in other settings [18, 58–62], and to design interventions to address them. The study authors are currently pursuing this through a bio-repository with maternal blood and urine samples collected during pregnancy and the postpartum period [63].

## Additional file

Additional file 1: **Table S1.** Adjusted Relative risk ratio : combining <6 months and 7-14 months. (DOCX 19 kb)

## Abbreviations

aRRR: Adjusted relative risk ratio
CHW: Community health worker
DHS: Demographic Health Survey
IOI: Inter-outcome interval
IPI: Inter-pregnancy interval
LBW: Low birth weight
SGA: Small for gestational age
WHO: World Health Organization

## References

[1] Bhattacharya S, Smith N (2011). Pregnancy following miscarriage: what is the optimum interpregnancy interval?. Womens Health (Lond Engl).

[2] Grisaru-Granovsky S (2009). Effect of interpregnancy interval on adverse perinatal outcomes--a national study. Contraception.

[3] Nabukera SK (2008). Interpregnancy interval and subsequent perinatal outcomes among women delaying initiation of childbearing. J Obstet Gynaecol Res.

[4] Nabukera SK (2009). Racial disparities in perinatal outcomes and pregnancy spacing among women delaying initiation of childbearing. Matern Child Health J.

[5] Norton M, Shelton JD (2011). Stillbirth and healthy timing and spacing of pregnancy. Lancet.

[6] Shelley J (2010). Miscarriage And Time To Next Pregnancy. BMJ.

[7] Yigzaw M, Enquselassie F (2010). Birth spacing and risk of child mortality at Kalu district South Wollo Zone of Amhara region Ethiopia. Ethiop Med J.

[8] Conde-Agudelo A (2005). Effect of the interpregnancy interval after an abortion on maternal and perinatal health in Latin America. Int J Gynaecol Obstet.

[9] Kozuki N, Walker N (2013). Exploring the association between short/long preceding birth intervals and child mortality: using reference birth interval children of the same mother as comparison. BMC Public Health.

[10] Kozuki N (2013). The associations of birth intervals with small-for-gestational-age, preterm, and neonatal and infant mortality: a meta-analysis. BMC Public Health.

[11] Raj A, McDougal L, Rusch ML (2014). Effects of young maternal age and short interpregnancy interval on infant mortality in South Asia. Int J Gynaecol Obstet.

[12] Houle B (2013). Household context and child mortality in rural South Africa: the effects of birth spacing, shared mortality, household composition and socio-economic status. Int J Epidemiol.

[13] Chen, I., G.S. Jhangri, and S. Chandra, Relationship between interpregnancy interval and congenital anomalies. Am J Obstet Gynecol, 2014. 210(6). 10.1016/j.ajog.2014.02.002 24508646

[14] Conde-Agudelo A (2005). Effect of the interpregnancy interval on perinatal outcomes in Latin America. Obstet Gynecol.

[15] Conde-Agudelo A, Rosas-Bermudez A, Kafury-Goeta AC (2006). Birth spacing and risk of adverse perinatal outcomes: a meta-analysis. JAMA.

[16] Zhu BP (1999). Effect of the interval between pregnancies on perinatal outcomes. N Engl J Med.

[17] Rutstein S, Winter R. The Effects of Fertility Behavior on Child Survival and Child Nutritional Status: Evidence from the Demographic and Health Surveys, 2006 to 2012. 2014. USAID. .

[18] USAID. USAID Maternal Health Strategy, 2014-2020.Toward Ending Preventable Maternal Mortality. 2014 [cited 2015 01 January 2014]; Available from: http://www.mchip.net/sites/default/files/mchipfiles/USAID%20MH%20Strategy%20Apr.22.14.pdf.

[19] Unicef. Monitoring the situation of women and children: current status and progress 2015 [cited 2016; Available from: http://data.unicef.org/topic/child-survival/neonatal-mortality/.

[20] WHO. Report of a technical consultation on birth spacing. 2005 13-15 June 2007; Available from: http://www.who.int/reproductivehealth/publications/family_planning/WHO_RHR_07_1/en/.

[21] DaVanzo J (2008). The effects of pregnancy spacing on infant and child mortality in Matlab, Bangladesh: how they vary by the type of pregnancy outcome that began the interval. Popul Stud (Camb).

[22] DaVanzo J (2007). Effects of interpregnancy interval and outcome of the preceding pregnancy on pregnancy outcomes in Matlab Bangladesh. BJOG.

[23] BDHS, Bangladesh Demographic and Health Survey 2011. 2011, Bangladesh Demographic and Health Survey Dhaka. p. 67.

[24] BDHS, Bangladesh Demographic and Health Survey 2015 page 57. 2014, Bangladesh Demographic and Health Survey. p. 56-57.

[25] Baqui AH (2011). Levels, timing, and etiology of stillbirths in Sylhet district of Bangladesh. BMC Pregnancy Childbirth.

[26] Nonyane BAS (2013). Clustering of Neonatal Deaths in Bangladesh: Results From the Projahnmo Studies. Paediatric and Perinatal Epidemiology.

[27] Baqui AH (2008). Effect of community-based newborn-care intervention package implemented through two service-delivery strategies in Sylhet district, Bangladesh: a cluster-randomised controlled trial. Lancet.

[28] Conde-Agudelo A (2012). Effects of birth spacing on maternal, perinatal, infant, and child health: a systematic review of causal mechanisms. Stud Fam Plann.

[29] Rutstein SO. Further evidence of the effects of preceding birth intervals on neonatal infant and under-five years mortality and nutritional status in developing countries: evidence from the Demographic and Health Surveys. USAID. 2008. 10.1016/j.ijgo.2004.11.012 15820369

[30] WHO. Maternal, newborn, child and adolescent health. 2015 [cited 2017; Available from: http://www.who.int/maternal_child_adolescent/epidemiology/stillbirth/en/.

[31] Vyas S, Kumaranayake L (2006). Constructing socio-economic status indices: how to use principal components analysis. Health Policy and Planning.

[32] Baqui AH (2018). Impact of integrating a postpartum family planning program into a community-based maternal and newborn health program on birth spacing and preterm birth in rural Bangladesh. J Global Health.

[33] Chandna A (2016). Inter-pregnancy interval and pregnancy outcomes, International Journal of Reproduction Contraception. Obstet Gynecol.

[34] Basso O, Olsen J, Christensen K (1998). Risk of preterm delivery, low birthweight and growth retardation following spontaneous abortion: a registry-based study in Denmark. Int J Epidemiol.

[35] Bentolila Y (2013). Effect of interpregnancy interval on outcomes of pregnancy after recurrent pregnancy loss. J Matern Fetal Neonatal Med.

[36] Love ER, Bhattacharya S, Smith NC (2010). Effect of interpregnancy interval on outcomes of pregnancy after miscarriage: retrospective analysis of hospital episode statistics in Scotland. BMJ.

[37] Goldstein RR, Croughan MS, Robertson PA (2002). Neonatal outcomes in immediate versus delayed conceptions after spontaneous abortion: a retrospective case series. Am J Obstet Gynecol.

[38] Schliep KC (2016). Trying to conceive after an early pregnancy loss; an assessment on how long couples should wait. Obstet Gynecol.

[39] Kangatharan C, Labram S, Bhattacharya S. Interpregnancy interval following miscarriage and adverse pregnancy outcomes: systematic review and meta-analysis. Hum Reprod Update. 2016. 10.1093/humupd/dmw043 27864302

[40] Rahman M, DaVanzo J, Razzaque A (2010). The role of pregnancy outcomes in the maternal mortality rates of two areas in Matlab Bangladesh. Int Perspect Sex Reprod Health.

[41] Davanzo, J., L. Hale, and M. Rahman, How long after a miscarriage should women wait before becoming pregnant again? Multivariate analysis of cohort data from Matlab, Bangladesh. BMJ Open, 2012. 2(4). 10.1136/bmjopen-2012-001591 PMC3425891 22907047

[42] de Weger, F.J., et al., Advanced maternal age, short interpregnancy interval, and perinatal outcome. Am J Obstet Gynecol, 2011. 204(5): p. 421 e1-9. 10.1016/j.ajog.2010.12.008 21288503

[43] Razzaque A (2005). Pregnancy spacing and maternal morbidity in Matlab, Bangladesh. Int J Gynaecol Obstet.

[44] Williams EK (2008). Birth interval and risk of stillbirth or neonatal death: findings from rural north India. J Trop Pediatr.

[45] Conde-Agudelo A (2012). Effects of birth spacing on maternal, perinatal, infant, and child Health: a systematic review of causal mechanisms. Studies in Family Planning.

[46] Khambalia A, O’Connor D, Zlotking S (2009). Periconceptional iron and folate status is inadequate among married women in rural Bangladesh. Journal of Nutrition..

[47] Lindstrom, E. and M.B. Hossain, Prevalence of anemia and micronutrient deficiencies in early pregnancy in rural Bangladesh: the MiniMat Trial, Acta Obstet Gynecol Scand. 2011; 90(1):47-56. doi: 10.1111/j.1600-0412.2010.01014.x. . Acta Obstet Gynecol Scand, 2011. **90**(1): p. 47-56. 10.1111/j.1600-0412.2010.01014.x 21275915

[48] Gaskins A (2014). Maternal prepregnancy folate intake and risk of spontaneous abortion and stillbirth. Obstet Gynecol.

[49] Megahed M, Taher IM (2004). Folate and homocysteine levels in pregnancy. British Journal of Biomedical Science. Brit J Biomed Sci.

[50] Pathak P (2004). Prevalence of multiple micronutrient deficiencies amongst pregnant women in a rural area of Haryana. Indian J Pediatr..

[51] Sabin KM (2003). Sexually transmitted infections prevalence rates in slum communities of Dhaka, Bangladesh. Int J STD AIDS..

[52] Ashrafunessa (2009). Seroprevalence of cytomegalovirus antibody in antenatal population in Bangladesh. Bangladesh Med Res Counc Bull,Letter to the Edito.

[53] Liesnard C (2000). Prenatal diagnosis of congenital cytomegalovirus infection: prospective study of 237 pregnancies at risk. Obstet Gynecol.

[54] Bristow BN (2011). Congenital cytomegalovirus in the United States. PLoS Negl Trop Dis.

[55] Usta A (2016). Screening cytomegalovirus infections in first trimester of gestation among high prevalence population. Acta Medica Anatoli.

[56] Fowler K, Stagno S, Pass R (2004). Interval between births and risk of congenital cytomegalovirus infection. Clin Infec Dis.

[57] Lehnertz NB (2013). Local understandings and current barriers to optimal birth intervals among recently delivered women in Sylhet District. Bangladesh. Int Health.

[58] Steenhoff AP (2008). Invasive pneumococcal disease among human immunodeficiency virus-infected children, 1989-2006. Pediatr Infect Dis J.

[59] Rutstein SO (2005). Effects of preceding birth intervals on neonatal, infant and under-five years mortality and nutritional status in developing countries: evidence from the demographic and health surveys. Int J Gynaecol Obstet.

[60] Williamson K (2008). Within-year differences in reproductive investment in laboratory zebra finches (Taeniopygia guttata), an opportunistically breeding bird. Naturwissenschaften.

[61] Wood SM (2008). Primary varicella and herpes zoster among HIV-infected children from 1989 to 2006. Pediatrics.

[62] Fotso JC (2013). Birth spacing and child mortality: an analysis of prospective data from the Nairobi urban health and demographic surveillance system. J of Biosoc Sci.

[63] Baqui AH (2017). Understanding biological mechanisms underlying adverse birth outcomes in developing countries: protocol for a prospective cohort (AMANHI bio-banking) study. J Glob Health.

