# Supplementary material for: Pregnancy intervals after stillbirth, neonatal death and spontaneous abortion and the risk of an adverse outcome in the next pregnancy in rural Bangladesh
Source: BMC Pregnancy Childbirth. 2019 Feb 9;19:62. doi: 10.1186/s12884-019-2203-0 (PMC6368961; doi:10.1186/s12884-019-2203-0)
Supplement: Supplementary file 1 — Table S1. Adjusted Relative risk ratio : combining <6 months and 7-14 months. (DOCX 19 kb) [file 12884_2019_2203_MOESM1_ESM.docx]

**Supplemental Table 1: Adjusted Relative risk ratio : combining <6 months and 7-14 months**

| **Preceding outcome=spontaneous abortion** | | | | |
| --- | --- | --- | --- | --- |
|  | **Adjusted Relative Risk Ratio** |  | **Lower CI** | **Upper CI** |
| Spontaneo**us abortion** |  |  |  |  |
| <=14 months | 2.70 |  | 1.49 | 4.90 |
| 15-26 months | 1.07 |  | 0.57 | 2.03 |
| **27-50 months** | **Reference** | | | |
| 51-74 months | 0.33 |  | 0.11 | 0.97 |
| 75-263 months | 0.28 |  | 0.05 | 1.58 |
|  |  |  |  |  |
| **Stillbirth** |  |  |  |  |
|  |  |  |  |  |
| <=14 months | 1.07 |  | 0.66 | 1.71 |
| 15-26 months | 0.80 |  | 0.49 | 1.32 |
| **27-50 months** | **Reference** | | | |
| 51-74 months | 1.66 |  | 0.74 | 3.74 |
| 75-263 months | 0.73 |  | 0.09 | 5.67 |
|  |  |  |  |  |
| **Neonatal death** |  |  |  |  |
|  |  |  |  |  |
| <=14 month | 1.55 |  | 0.97 | 2.46 |
| 15-26 months | 1.17 |  | 0.72 | 1.90 |
| **27-50 months** | **Reference** | | | |
| 51-74 months | 1.09 |  | 0.42 | 2.82 |
| 75-263 months | 1.20 |  | 0.26 | 5.61 |
|  |  |  |  |  |
| **Preceding outcome=Stillbirth** | | | | |
|  | **Adjusted Relative Risk Ratio** |  | **Lower CI** | **Upper CI** |
| **Spontaneous abortion** |  |  |  |  |
| <=14 months | 2.53 |  | 1.19 | 5.36 |
| 15-26 months | 0.77 |  | 0.36 | 1.66 |
| **27-50 months** | **Reference** |  |  |  |
| 51-74 months | 0.37 |  | 0.07 | 2.03 |
| 75-263 months | 0.83 |  | 0.09 | 7.38 |
|  |  |  |  |  |
| **Stillbirth** |  |  |  |  |
| <=14 months | 1.91 |  | 1.33 | 2.75 |
| 15-26 months | 1.32 |  | 0.92 | 1.91 |
| **27-50 months** | **Reference** | | | |
| 51-74 months | 1.12 |  | 0.54 | 2.34 |
| 75-263 months | 1.02 |  | 0.34 | 3.01 |
|  |  |  |  |  |
| Neonatal death |  |  |  |  |
| <=14 month | 1.44 |  | 0.88 | 2.36 |
| 15-26 months | 1.29 |  | 0.79 | 2.09 |
| **27-50 months** | **Reference** | | | |
| 51-74 months | 0.99 |  | 0.36 | 2.72 |
| 75-263 months | 1.34 |  | 0.37 | 4.77 |
|  |  |  |  |  |
| **Preceding outcome=Neonatal Death** | | | | |
|  | **Adjusted Relative Risk Ratio** |  | **Lower CI** | **Upper CI** |
| Spontaneous **abortion** |  |  |  |  |
| <=14 months | 2.82 |  | 1.46 | 5.45 |
| 15-26 months | 1.58 |  | 0.80 | 3.11 |
| **27-50 months** | **Reference** | | | |
| 51-74 months | 1.09 |  | 0.28 | 4.20 |
| 75-263 months | 1.97 |  | 0.49 | 7.91 |
|  |  |  |  |  |
| **Stillbirth** |  |  |  |  |
| <=14 months | 3.27 |  | 1.94 | 5.51 |
| 15-26 months | 2.32 |  | 1.38 | 3.91 |
| **27-50 months** | **Reference** | | | |
| 51-74 months | 1.02 |  | 0.33 | 3.11 |
| 75-263 months | 4.02 |  | 1.56 | 10.36 |
|  |  |  |  |  |
| Neonatal death |  |  |  |  |
| <=14 month | 2.82 |  | 2.07 | 3.85 |
| 15-26 months | 1.70 |  | 1.24 | 2.33 |
| **27-50 months** | **Reference** | | | |
| 51-74 months | 0.54 |  | 0.23 | 1.28 |
| 75-263 months | 1.14 |  | 0.43 | 3.00 |
